# Editorial: The Use of Nanoparticles in the Diagnosis and Therapy of Infectious Disease in Animals

**DOI:** 10.3389/fvets.2021.829540

**Published:** 2021-12-24

**Authors:** Renata K. T. Kobayashi, Santosh Dhakal, Gerson Nakazato

**Affiliations:** ^1^Department of Microbiology, Center of Biological Sciences, State University of Londrina, Londrina, Brazil; ^2^W. Harry Feinstone Department of Molecular Microbiology and Immunology, The Johns Hopkins Bloomberg School of Public Health, Baltimore, MD, United States

**Keywords:** nanotechnology, microbial pathogens, resistance, detection, molecular methods

The infectious diseases are extremely relevant to veterinary medicine since they are responsible for most appointments in the veterinary hospitals and clinics, extensive economic losses in animal production, along with the potential zoonotic risk of many of these etiological agents. Each disease has different sources of infection, transmission routes, entry points and possible susceptible animals, requiring efficient diagnosis and treatments.

It is also essential to reinforce the “One Health” concept, which aims at the inseparability of human, animal, and environmental health. Numerous epidemics and pandemics that originate from livestock, including the influenza virus and coronaviruses, highlight the importance of early diagnosis, continuous monitoring, and efficient prevention of the emerging or re-emerging animal infectious pathogens to minimize their significant impacts on the animal health, food safety and food security, and public health (1).

Nanotechnology has revolutionized the field of infectious disease diagnosis, and the development of therapeutics and preventatives. Their significance in biomedical application is due to their smaller size and unique physicochemical properties which allows for the controlled release of the drugs, targeted drug delivery, and *in vivo* immunomodulation. Nanotechnology has been used in various aspects of veterinary medicine including disease diagnosis, treatment, development of adjuvants and vaccines, drug delivery, and solving problems related to animal nutrition and reproduction (2). Nanotechnology provides more efficient diagnostic tools and therapies, whether in terms of sensitivity, specificity, speed, or cost. Various nanomaterials have been used in veterinary diagnostics and therapeutics including metal nanoparticles, polymeric nanoparticles, nanoemulsions, liposomes, and nanocrystals (2). The use of nanotechnology in veterinary medicine will continue in the future leading to advancements in the diagnosis and therapies of infectious diseases in animals safeguarding both animal and human health.

The aim of this Research Topic was to bring together the use of nanotechnology in the detection of veterinary infectious pathogens (bacteria, viruses, protozoa, and fungi) and in animal infectious disease management (therapies and vaccinations). This Research Topic includes four articles in which three describe diagnostic approaches and one highlights application of nanoparticles as antimicrobial agent for the veterinary diseases. These studies showed the advances of nanotechnology in the veterinary field, mainly for the diagnosis of emerging diseases. There is a large potential of these nanoparticles in the development of new products and processes to detect, prevent and eliminate pathogens from animal sources. In a review, Manhas et al. highlighted latest updates on the nanomaterials-based diagnostic tests to six emerging/re-emerging poultry and livestock diseases namely avian influenza (colloidal particle-conjugated antibodies), post-weaning multisystemic wasting syndrome (antibody-modified gold-platinum and silica dioxide nanospheres), Newcastle disease (iron oxide nanoparticles–magnetic separation), anthrax (biosensor with single-stranded modified-AuNPs probes), brucellosis (oligonucleotide-modified AuNP-based colorimetric assay) and aflatoxicosis (aptasensor with Au nanowires/graphene oxide). They reported that immuno-based and molecular-based functionalization and alterations of various nanomaterials have improved the speed of pathogens and toxins detections with superior sensitivity and specificity. Ge et al. developed blue silica nanoparticles (SiNPs)-based lateral flow immunoassay (LFIA) for rapid detection of human brucellosis with high sensitivity and specificity. For this, *Staphylococcal* protein A (SPA) and lipopolysaccharide of *Brucella* spp. were used in design of SiNPs-based LFIA that could detect antibody target on serum samples. Using brucellosis positive and negative human serum samples, Ge et al. showed 87% and 93.9% positive and negative predictive values to this assay which can be used for on-site diagnosis of the pathogen.

A molecular strategy to express the capsid protein (Cap) of Porcine Circovirus Type 4 (PCV4) based on *Escherichia coli* expression system was approached in the study of Wang et al. The PCV4 virus-like particles (VLPs) were of size ~20 nm and had high antigenicity. As these nanostructures have unique morphology and immunogenicity they can be used for serological diagnostics and for vaccine development in the future.

Water disinfection is very important for public health and several diseases may come from water sources. Zhang et al. showed that copper/carbon core/shell nanoparticles (CCCSNs) and a commercial CCCSNs filter product were efficient against *Saprolegnia parasitica* which is one of the most prevalent oomycete diseases in aquaculture and important pathogen of finfish. Interestingly, these nanoparticles serve as an alternative for formalin in water treatment, corroborating for prevention and control of *S. parasitica*.

Studies included in this Research Topic demonstrated that nanotechnology needs more exploration in the field of veterinary medicine, mainly in production animals, because the results of these four articles showed a great potential to generate new products using nanoparticles for the diagnosis, treatment and prevention of veterinary infectious diseases. It is noteworthy that early detection or prevention of animal diseases not only protects animal health and ensure animal welfare but also provides food security and protects human health. Thus, nanotechnology should be explored fully for the development of diagnostic tools, therapeutics, and vaccines to protect animal health and hence the public health.

## Author Contributions

All authors listed have made a substantial, direct, and intellectual contribution to the work and approved it for publication.

## Funding

This work was supported in part by the National Council for Scientific and Technological Development (CNPq)–processes 433656/2018-2 Call MCTIC/CNPq 28/2018 and 313305/2019-6 to RK and CNPq–process 315435/2018-6 to GN.

## Conflict of Interest

The authors declare that the research was conducted in the absence of any commercial or financial relationships that could be construed as a potential conflict of interest.

## Publisher's Note

All claims expressed in this article are solely those of the authors and do not necessarily represent those of their affiliated organizations, or those of the publisher, the editors and the reviewers. Any product that may be evaluated in this article, or claim that may be made by its manufacturer, is not guaranteed or endorsed by the publisher.
